# Compliance With the World Health Organization Surgical Safety Checklist at a Tertiary Care Hospital: A Closed Loop Audit Study

**DOI:** 10.7759/cureus.39808

**Published:** 2023-05-31

**Authors:** Hamza K Toru, Zahid Aman, Muhammad Haider Ali, Waqas Kundi, Muhammad A Khan, Fawad Ali, Shandana Khan, Muhammad J Zahid, Zaka Ullah Jan

**Affiliations:** 1 General Surgery, Khyber Teaching Hospital, Peshawar, PAK; 2 General Surgery, Hayatabad Medical Complex, Peshawar, PAK; 3 Surgery, Hayatabad Medical Complex, Peshawar, PAK

**Keywords:** closed loop, audit, surgical safety, who, compliance

## Abstract

Background and objective: The WHO launched the "Safe Surgery Saves Lives" campaign in 2008 to improve patient safety during surgery. The campaign includes the use of the WHO Surgical Safety Checklist, which has been proven effective in reducing complications and mortality rates in several studies. This article discusses a clinical audit at a tertiary healthcare facility that assesses compliance with all three components of the checklist to minimize errors and improve safety standards.

Materials and methods: This prospective, observational, closed-loop clinical audit study was conducted at Hayatabad Medical Complex, a tertiary care public sector hospital located in Peshawar, Pakistan. The audit aimed to assess compliance with the WHO Surgical Safety Checklist. The first phase of the audit cycle commenced on October 5, 2022, and involved collecting data from 91 surgical cases in randomly selected operating rooms. Following the completion of the first phase on December 13, 2022, an educational intervention was then conducted on December 15 to underscore the significance of adhering to the checklist, and the second phase of data collection began the following day, ending on February 22, 2023. The results were analyzed using SPSS Statistics version 27.0.

Results: The first phase of the audit showed that there was poor compliance with the latter two parts of the checklist. Certain components of the WHO Surgical Safety Checklist were well-complied with, including patient identity confirmation (95.6%), obtaining informed consent (94.5%), and counting of sponges and instruments (95.6%), while the lowest compliance rates were in recording allergies (26.3%), assessing blood loss risk (15.3%), introducing team members (62.6%), and inquiring about patient recovery concerns (64.8%, 34%, and 20.8% for surgeons, anesthetists, and nurses, respectively). In the second phase, after an educational intervention, compliance with the checklist improved significantly, particularly for those components with low compliance rates in the first phase, marking recording allergies (89.0%), introducing team members 91.2%), and inquiring about patient recovery concerns (79.1%, 73.6%, and 70.3% for surgeons, anesthetists, and nurses, respectively).

Conclusion: The study showed that education is a critical factor in improving compliance with the WHO Surgical Safety Checklist. The study suggests that overcoming the obstacles to implementing the checklist requires a collaborative environment and effective instruction. It emphasizes the importance of adhering to the checklist in all surgical settings.

## Introduction

Surgery plays a crucial role in the modern healthcare setup by providing solutions to a wide range of medical conditions. Be it lifesaving emergencies or planned elective operations, surgery has the potential to alleviate patients’ suffering and improve their quality of life. Unfortunately, however, there have been many instances where despite the best intentions, surgery has resulted in adverse outcomes. A systematic review conducted over numerous studies from nine countries evaluating the occurrence of adverse incidents among patients undergoing surgery revealed that the majority of negative incidents were preventable if improvement endeavors are made to encompass not just mistakes in surgical technique but also non-operative measures [1].

In the early 2000s, it was made increasingly clear that there was a dire need for a standardized safety protocol to be adopted as a means of improving patient safety and outcome. As a response, WHO launched its global campaign "Safe Surgery Saves Lives" in 2008 to increase awareness regarding the importance of surgical safety and reduce the incidence of surgical deaths and complications [2]. To achieve this goal, the WHO Surgical Safety Checklist was devised and designed to be used in the operating room to ensure that critical safety procedures are followed consistently for every patient.

The checklist approach was adopted as it has many benefits: It aids in remembering tasks, particularly mundane ones that are easily forgotten in a pressure situation, prompts teamwork, and clearly establishes the minimum steps of an otherwise complicated process, thus, reducing errors, improving safety, and improving outcomes [3]. The checklist breaks down the surgical procedure into three distinct phases, each of which corresponds to a specific timeframe in the standard sequence of events: Sign-in, the period prior to the administration of anesthesia; Time-out, the period following anesthesia induction but before the actual incision; and Sign-out: the period after wound closure but before the patient leaves the operation theatre. The checklist is designed in such a way as to promote collaboration between the nursing team, anesthetists, and surgeons. Due to the emphasis on its verbal component, it prevents communication failures among the theatre staff who work collectively to ensure the safety of the patient during the procedure and reduce complications [2,4].

Numerous studies from all over the world have established that the implementation of the WHO Surgical Safety Checklist has resulted in noteworthy declines in both morbidity and mortality [4,5]. The objective of this clinical audit was to assess the conformity in our tertiary healthcare facility to all three components of the WHO Surgical Safety Checklist, with the ultimate goal of minimizing the occurrence of surgical complications and errors for future surgical patients.

## Materials and methods

This prospective, observational, closed-loop clinical audit was conducted at the Department of General Surgery at Hayatabad Medical Complex from October 2022 to February 2023. Hayatabad Medical Complex is a distinguished tertiary care public sector hospital located in Peshawar, Pakistan. After obtaining ethical approval from the IRB Board (approval number 234/IRB/HMC), the first phase of the audit cycle commenced on October 5, 2022, until December 14, 2022. Prior to beginning data collection, the lead auditor trained the team members regarding the appropriate utilization of the checklist to ensure adequate understanding when collecting data. A sample size of 91 patients who consented and underwent any surgery during the time duration of the audit was chosen for each phase of the audit cycle using non-probability consecutive sampling.

Hayatabad Medical Complex has 13 primary operating rooms, each assigned a unique number from 1 to 13. For the purpose of the study, to select the operating rooms for data collection, a random number generator was employed daily to randomly select two operating rooms. A structured questionnaire was then used to gather data, encompassing inquiries regarding the patient's biodata and the inclusion of all components of the WHO Surgical Safety Checklist. To minimize potential bias in data collection, the team working in the relevant operating rooms was not informed that they were being audited during the first phase of the study. Despite the fact that the components of the WHO Surgical Safety Checklist are typically verbalized aloud, they were also evaluated for performance based on documentation or clear observation.

After the conclusion of the initial phase of the audit cycle, an educational intervention was convened on December 15, 2022. The intervention took the form of a multi-disciplinary team meeting, comprising members from the nursing, anesthesia, and surgical departments. A presentation was delivered emphasizing the role of the WHO Surgical Safety Checklist as a tool for enhancing safety and underscoring the significance of adhering to it. Attendees were furnished with copies of the checklist and received instructions regarding the implementation of the checklist and the importance of using verbal communication. Subsequent to the intervention, the WHO Surgical Safety Checklist was officially integrated into the hospital's patient-file system, and efforts were made to enhance awareness regarding its utilization.

The second phase of the audit cycle was initiated following the educational intervention on the following day and continued from December 16, 2022, through February 22, 2023. During the first three days, members of the second phase, audit team members, served as checklist coordinators in multiple surgeries to provide a hands-on demonstration to the department on the proper utilization of the Surgical Safety Checklist, as was discussed during the educational intervention. Formal data collection for the second phase began after this period, utilizing the same structured questionnaire as employed during the first phase. Following data collection, SPSS Statistics version 27.0 (IBM Corp. Released 2020. IBM SPSS Statistics for Windows, Version 27.0. Armonk, NY: IBM Corp) was employed to analyze the results.

**Figure 1:**
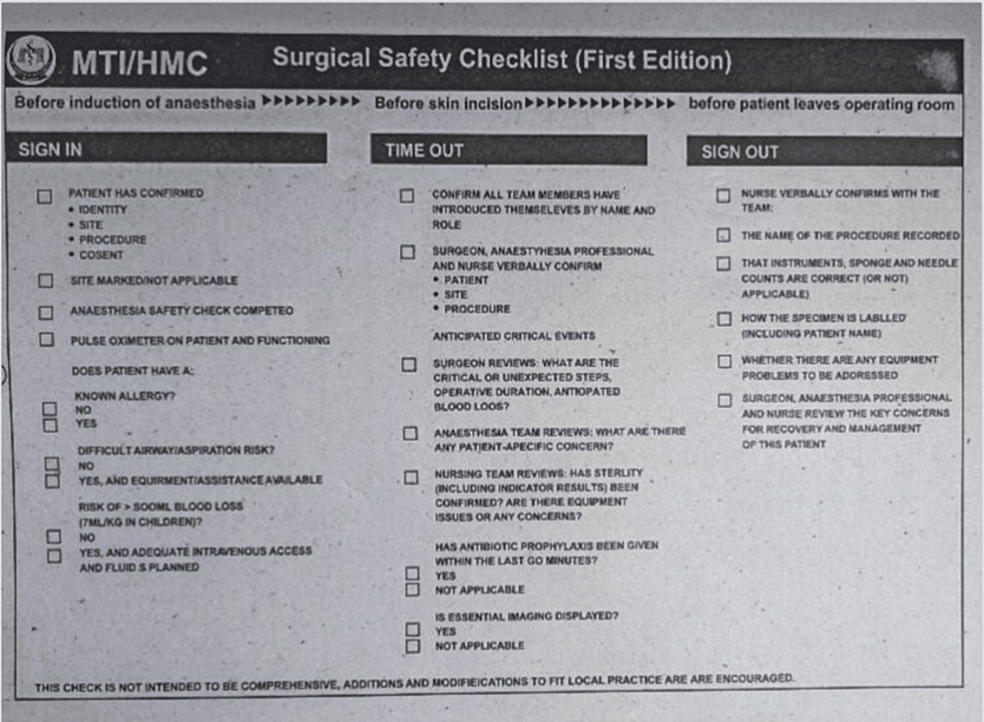
WHO Surgical Safety Checklist incorporated into the records of patients at Hayatabad Medical Complex

## Results

In the first phase of the audit cycle, a total of 91 surgical operations were observed. Several components of the checklist exhibited excellent compliance even in the initial phase, notably the confirmation of patient identity, obtaining informed consent, and the counting of sponges and instruments, which were marked in over 80% of the cases.

The lowest compliance rates were observed in recording allergies, assessment of blood loss risk, introducing team members, and inquiring about patient recovery concerns, all of which were marked in less than 40% of the cases. The remaining components of the Surgical Safety Checklist displayed an average compliance rate ranging from 50% to 70%.

Subsequent to the educational intervention, a total of 96 surgical procedures were observed in the second phase of the audit cycle, revealing a significant enhancement in compliance with the WHO Surgical Safety Checklist. The components that exhibited the lowest compliance rates in the first cycle displayed the most notable improvement, with an increase of over 50%. Specifically, the recording of allergies, introduction of team members, and inquiry regarding patient recovery concerns were marked in over 80% of the surgical cases observed in the second phase. The components that exhibited the highest compliance rates were the confirmation of patient identity (100%), the counting of sponges and instruments (98.9%), and obtaining informed consent (97.8%). Nevertheless, there was only a slight improvement in compliance rates observed for the assessment of blood loss risk, which increased from 15.1% in the first phase to 34.0% in the second phase. Similarly, the inquiry regarding key concerns post-operation by the surgeon demonstrated a modest increase of approximately 10%, from 64.8% in the first phase to 74.7% in the second phase.

**Table 1:** Compliance with the WHO Surgical Safety Checklist

	First Phase	Second Phase
Sign in	N(%)	N(%)
Identity confirmed	95.6	100.0
Site marked	72.5	92.3
Procedure confirmed	100	100
Consent given	94.5	97.8
Anesthesia checks complete	78.0	83.3
Pulse oximeter functioning	86.8	93.4
Known allergy	26.3	89.0
Difficult airway	78.0	87.9
Risk of > 500ml blood loss	15.3	34.0
Overall	71.9	86.4
Time out		
Team members introduction	62.6	91.2
Confirmation of patient’s identity, site, procedure	91.2	96.7
Antibiotic prophylaxis	56.0	88.3
Critical events anticipation: surgeon	65.9	92.3
Critical events anticipation: anesthetist	74.9	94.6
Critical events anticipation: nursing team	65.9	89.0
Essential imaging displayed	57.1	90.1
Overall	67.7	91.7
Sign out		
Name of procedure confirmed	98.9	100.0
Instrument, sponge, and needle count	95.6	98.9
Specimen labeled	92.3	95.6
Equipment problems	26.3	81.3
Key concerns for recovery: surgeon	64.8	79.1
Key concerns for recovery: anesthetist	34.0	73.6
Key concerns for recovery: nurse	20.8	70.3
Overall	61.8	85.5

## Discussion

WHO developed the Surgical Safety Checklist as a means to enhance patient safety [2]. However, in our establishment, there existed an insufficiency of awareness regarding the formal implementation of the checklist in our operating rooms. Thus, the principal objective of our quality improvement project was to assess the degree of adherence to the components of the WHO Surgical Safety Checklist within our present practice and to raise awareness regarding its utilization. The ultimate aim of this project was to ascertain any potential enhancement in compliance subsequent to our educational intervention, with the ultimate objective of advancing the standards of patient safety.

The outcomes of the initial phase of our audit cycle indicated that with the exception of two components, there was decent compliance with most of the elements included in the "sign-in" section of the Surgical Safety Checklist; however, compliance with the "time-out" and "sign-out" part was significantly lower. These findings are similar to a study conducted in Lahore in 2022 which also exhibited lower compliance with the "time-out" and "sign-out" part of the checklist as compared to "sign-in" [6]. The observed disparity in compliance rates between the first and subsequent sections of the checklist may be attributed to the documental nature of the former, as opposed to the active verbal communication required by the latter two sections among members of the surgical team in the operating theatre. In our study too, we observed that there was relatively higher compliance with the documental components of the checklist compared to the verbal. This is in accordance with a study done in Sweden which showed low compliance with the "time-out" part of the audit, inferring that those items of the checklist that encourage communication between the theatre staff are poorly applied [7]. This aspect presents an opportunity for improvement, as multiple observational studies have demonstrated a correlation between proficient communication, cohesive teamwork, and their positive impact on patient safety [8]. The two components of the "sign-in" phase that exhibited inadequate compliance rates were the inquiry about a known allergy at 26.3% and the assessment for the risk of more than 500 ml blood loss at 15.3%. Although inquiring about the patient's allergy history was typically carried out during the initial admission phase, there were minimal instances in which an allergy history was verified before commencing surgery. It is critical to inquire about allergies before surgery to enable the theater staff to be aware of them and avoid administering any drugs that may cause an adverse reaction, given that up to 40% of patients undergoing surgery have a positive history of allergy [9]. The compliance rate for the assessment of bleeding risk in the "sign-in" phase was notably low, as it was frequently deemed unimportant by the surgeon, particularly in the case of apparently healthy individuals undergoing elective surgery. However, this is inconsistent with the recommendations of the British Committee for Standards in Hematology, which stipulates that a comprehensive bleeding history should be obtained from every patient before undergoing surgery [10].

Upon conclusion of the initial phase of the audit cycle, an educational intervention was convened by the audit team to review the findings. During this meeting, a comprehensive analysis of the low compliance observed in the first phase was conducted. The deliberation highlighted the primary contributing factors to the low compliance, namely inadequate awareness regarding the WHO Surgical Safety Checklist, ineffective communication among theater staff, and a general lack of recognition regarding the checklist's purpose and benefits. In our literature search, we came across studies that recognized similar reasons for a low level of compliance with the checklist. A study conducted in Washington observed that a major cause of such low compliance was poor recognition among theatre staff concerning the purpose and benefits of the checklist, while a local study conducted in Rawalpindi agreed with our findings that low compliance was particularly evident with those components of the checklist that required verbal communication between the different team members as opposed to the documentation ones [11,12].

Following the intervention, additional awareness was spread concerning the utilization of the WHO Surgical Safety Checklist among all surgical units. Furthermore, the WHO Surgical Safety Checklist was formally incorporated into the patient file system to ensure easy accessibility and to serve as a prompt for theatre staff. This approach is akin to a study that employed wall-mounted charts as a reminder for the surgical team, resulting in a significant improvement in compliance with the checklist [13]. Our study found that there was a significant improvement in compliance with the WHO Surgical Safety Checklist in the second phase of our audit cycle, showing the positive impact of the educational intervention. The most notable improvement was observed in the "time-out" and "sign-out" sections of the checklist, which previously had lower compliance rates, and overall more than 85% compliance was successfully achieved in all three parts of the checklist. This underscores the impact of a straightforward and cost-effective measure, such as introducing a checklist in a multidisciplinary setting, in significantly enhancing the safety and quality of surgical procedures in operating theaters and reducing the incidence of adverse surgical outcomes. In addition, it promotes a culture of collaboration and effective communication, aimed at accomplishing tasks that ultimately lead to positive outcomes for the patient [14].

Our quality improvement closed-loop audit study was successful in implementing positive outcomes by enhancing the quality of care provided to our patients and fostering an environment where clinicians strive to deliver optimal care that aligns with established standards. However, it is important to acknowledge the limitations of our study, which are inherently localized and may not be readily applicable to other tertiary care hospitals within our region. Despite this, our study can serve as a blueprint for similar audits in other major healthcare institutions and eventually prompt the national healthcare regulatory authorities to use their influence in the implementation of the WHO Surgical Safety Checklist nationwide. Our study also unearthed certain structural impediments with the process of quality improvement audits such as the strict hierarchical structure of our department. This was especially evident during the “time-out” phase where the break taken by the checklist coordinator who was often a nurse of a junior trainee was often not taken too well by the consultant surgeon, a picture similar to what was observed by a study conducted in 2018 [15]. To overcome these obstacles, it is crucial to cultivate a conducive learning environment that underscores the significance of the checklist in mitigating adverse surgical outcomes and elevating safety standards. In addition, following the recommendations of WHO [2], appropriate adjustments should be made to the checklist over time to align with local practices, thereby facilitating a seamless transition to a more collaborative and safer surgical setting.

## Conclusions

Our study demonstrated that a comprehensive educational intervention can substantially enhance compliance with the WHO Surgical Safety Checklist, which is a crucial tool for minimizing post-operative complications. It is imperative to implement and adhere to the checklist in all surgical settings, and barriers to its adoption can be overcome by fostering a collaborative environment and delivering effective instruction.
